# A serverless computing architecture for Martian aurora detection with the Emirates Mars Mission

**DOI:** 10.1038/s41598-024-53492-4

**Published:** 2024-02-06

**Authors:** David Pacios, José Luis Vázquez-Poletti, Dattaraj B. Dhuri, Dimitra Atri, Rafael Moreno-Vozmediano, Robert J. Lillis, Nikolaos Schetakis, Jorge Gómez-Sanz, Alessio Di Iorio, Luis Vázquez

**Affiliations:** 1 Facultad de Informática, Universidad Complutense de Madrid, Calle del Prof. José García Santesmases, 9, 28040 Madrid, Spain; 2 Center for Space Science, New York University Abu Dhabi, Saadiyat Marina District, Abu Dhabi, 129188 Abu Dhabi UAE; 3 University of California, Gauss Way, 7, Berkeley, CA 94720 USA; 4 School of Production Engineering and Management, Computational Mechanics and Optimization Laboratory, Technical University of Crete, University Campus, Akrotiri, 73100 Chania, Greece; 5 Alma Sistemi Srl, University of California, Via dei Nasturzi n.4, 00012 Guidonia Montecelio, Lazio Italy

**Keywords:** Computer science, Information technology, Atmospheric dynamics, Computational astrophysics

## Abstract

Remote sensing technologies are experiencing a surge in adoption for monitoring Earth’s environment, demanding more efficient and scalable methods for image analysis. This paper presents a new approach for the Emirates Mars Mission (Hope probe); A serverless computing architecture designed to analyze images of Martian auroras, a key aspect in understanding the Martian atmosphere. Harnessing the power of OpenCV and machine learning algorithms, our architecture offers image classification, object detection, and segmentation in a swift and cost-effective manner. Leveraging the scalability and elasticity of cloud computing, this innovative system is capable of managing high volumes of image data, adapting to fluctuating workloads. This technology, applied to the study of Martian auroras within the HOPE Mission, not only solves a complex problem but also paves the way for future applications in the broad field of remote sensing.

## Introduction

Remote sensing technologies have become an essential tool for monitoring the Earth’s environment, providing valuable insights into natural disasters, climate change, land use, and more. With the growing availability of high-resolution satellite and aerial imagery, there is a need for efficient and scalable image analysis methods to process and extract information from this vast amount of data. Traditional approaches to image analysis often require significant computational resources, making them impractical for large-scale data processing.

Image analysis knowledge is usually coded in form of programs that need extensive computational resources. Image analysis is one of the areas where cloud computing alternatives are being considered. Mainly because of its requirement of a number of GPUs needed to perform computations, something that has not changed much in the last decade^1^. However, lack of experience in determining the best could architecture can result into additional costs when compared with local computing approaches^2^.

Recent advances in cloud computing and serverless architectures have opened up new possibilities for scalable and cost-effective image analysis. Serverless computing enables developers to focus on the application logic without worrying about infrastructure management, while cloud computing provides affordable, low cost computing resources for data processing^3^.

In this study, we shall address the question whether it is possible to perform such affordable low-cost analysis on real cases. This paper demonstrates that it is possible, providing benchmarks and proposing a serveless architecture for image analysis that leverages the power of OpenCV and machine learning algorithms, utilizing data from the Emirates Mars Mission’s HOPE probe.

This architecture is designed to perform image classification, object detection, and segmentation in a fast and cost-effective way. It uses OpenCV, a popular computer vision library, for image processing tasks such as noise reduction, thresholding, and edge detection. It also incorporates machine learning algorithms for tasks such as object detection and segmentation. The architecture is built on top of AWS Lambda, a serverless computing service, and AWS S3, a scalable and durable storage service. It leverages the scalability and elasticity of cloud computing to handle large volumes of image data and adapt to changing workloads.

To evaluate the performance of our architecture, experiments were conducted using a dataset of 200 images. The OpenCV classification and the machine learning inference performance was measured using AWS CloudWatch.

The results included in this paper show that this serverless architecture is capable of analyzing numerous images in a short amount of time while using minimal resources. The OpenCV classification completed very quickly, indicating that the system is well-suited for real-time analysis of images. The machine learning inference took longer than the OpenCV classification, but still performed well given the complexity of the task.

The paper is organized as follows. In “Martian auroras and the hope mission” section is an introduction to the problem of remote sensing in the case of the HOPE mission, i.e. image analysis for aurora detection. In "Cloud and serverless computing for data science" section describes cloud computing solutions for data science research. In "Proposed serverless architecture" section presents a serverless architecture, including AWS Lambda preprocessing and classification functions, and the adaptations that were necessary for the image analysis problem. In "Machine Learning" section describe the machine learning methods used. In "Experiments and results" section presents experimental results that illustrate the cost and duration of the computation for the experiments. In "Discussion" section contains discussion and conclusions.

## Martian auroras and the hope mission

Since their discovery in 2005, Martian auroras have been of great interest to the planetary science and space physics communities. They reveal the complex dynamics between the Martian atmosphere, solar wind and the unique “hybrid” magnetosphere of the planet (so-called because it shares attributes with both induced magnetosphere such as Venus and intrinsic magnetosphere such as the Earth or Jupiter^4^). Three categories of auroras have been observed on Mars so far – nightside discrete auroras resulting from suprathermal (10s eV to  keV) electron precipitation, nightside diffuse auroras resulting from high-energy electrons and protons (hundreds of keV), and proton auroras, which as the name suggests are caused by solar wind protons directly depositing energy in the dayside atmopshere causing auroral emissions. In the following paragraphs, we will describe these auroras only briefly and would refer the readers to a review paper for more details^5^.

Due to the lack of a global magnetic field, discrete auroras are not confined to the Martian polar regions (like Earth), but can appear anywhere the crustal magnetic fields weak and/or mostly vertical^6,7^, i.e. anywhere the magnetic topology allows electrons to access the atmosphere. Discrete auroras are distinct from diffuse auroras, which are more spread out and do not have well-defined boundaries. On Mars, it is understood that diffuse auroras are caused by the precipitation of high-energy electrons and protons^8^. The proton auroras occur mainly on the dayside of Mars and are excitations of the solar wind protons depositing in the Mars atmosphere as energetic neutral atoms (ENAs) of hydrogen^9^.

The Emirates Mars Mission (EMM) or “Hope” is the UAE’s spacecraft, operating in Martian orbit since February 2021^10,11^. It has an orbital period of $\sim$ 55 hours with an apogee of 42,650 km and a perigee of 19,970 km. Because of its unique orbit, it is able to observe all geographic regions within 72 hours and most latitudes and local times in 4 orbits or about 9 days, and is therefore a great tool to study phenomena occuring on a global scale on the planet^12^. The Emirates Mars Ultraviolet Spectrometer (EMUS) instrument on board the orbiter is a UV spectrometer capable of observing in the 100–170 nm wavelength range^13^. Due to these unique capabilities, auroras have been observed synoptically in the far-UV (FUV) for the first time on Mars with this instrument. In particular, discrete **electron** auroras have been observed to be brightest in the 130.4 nm oxygen line, which can be seen in about three fourth of all of EMUS observations. This oxygen line is produced by the de-excitation of the 2p4 (3P) state to the 2p3 (5S) ground state. Coincident obeservations of the discrete electron auroras are also found in the oxygen 135.6 nm line, although these are relatively weak and noisy. On rare occasions, observations of extreme proton aurora are found in 102.7 and 121.6 nm hydrogen Lyman-$\beta$ and Lyman-$\alpha$ respectively. These proton aurora occur on the dayside unlike the nightside discrete electron auroras discussed above. Other observations in the 98.9 and 104 nm are extremely noisy and weak.

Along with the unprecedented frequency of aurora observation, EMUS have also revealed new types of discrete auroras that can occur in regions without significant crustal magnetic fields as well as auroras with new morphology, such as sinuous discrete auroras that extend thousands of kilometers across the nightside^14^. So far these observations have no unambiguous explanation and work is ongoing to understand the physics driving these emissions, including comparisons with in situ data from the MAVEN spacecraft^15^. These recent observations are unexpected and the physics underpinning these mechanisms in not known as of now. These new discoveries present an unprecedented opportunity to characterize auroras on Mars in terms of their occurrence rates and mechanism of excitations, and thereby improve our understanding of the solar wind interaction with Mars.

In order to better understand this new phenomenon, there is a need for an automated detection algorithm which can identify these auroras and provide with details such as its location, size, shape, and intensity. To that end, our work presents an automated technique to detect these electron auroras on Mars observed **abundantly** in O 130.4 nm emissions via EMUS.

We describe the method in the following sections.

## Computational solution

### Cloud and serverless computing for data science

Cloud computing^16^ has become an increasingly popular choice for data science projects^17,18^, as it offers a number of advantages over traditional on-premises computation infrastructures, including the following: (i) * scalability*, cloud computing provides the ability to scale resources up or down as needed, allowing data scientists to quickly and easily scale their projects as the data grows; (ii) *cost-effectiveness*, the pay-as-you-go model used in cloud computing makes it more accessible for small and medium-sized organizations to adopt data science projects; (iii) *high availability*, cloud computing services provide different high availability and automatic failover solutions, which ensures that data science projects are available and accessible even in the event of a failure; (iv) *flexibility*, allowing data scientists to work with a variety of programming languages, frameworks, and tools; and (v) *access to a wide range of tools and services* offered by cloud computing platforms that can be used for data science projects, such as data storage, data processing, machine learning, and big data.

Main costs in CC comes from two elements: computing time and storage. Traditional approaches require the creation of virtual machines that are running constantly, a very basic IaaS (Infrastructure as a Service) approach. This kind of solution implies the highest costs, because it precisely requires constant CPU/GPU time and storage for the machine itself.

More recently, a new cloud computing service model has been delivered, the Serverless model^19,20^, which represents a paradigm shift in the way users build and deploy their applications. In serverless computing, the provider is responsible for executing a piece of code, by dynamically allocating the resources, and scaling on demand. This eliminates the need for manual scaling and the associated costs of maintaining idle resources. In this model, the user is only charged for the specific resources and the exact amount of time used to run their code^21,22^, which can result in cost savings for the user. Serverless computing also allows for increased development agility, as it allows developers to focus on writing and deploying code without having to worry about managing and maintaining the underlying infrastructure. In a serverless environment, the application logic is commonly implemented as a set of stateless functions that are triggered by events (e.g., API calls, message queues or scheduled tasks), and are executed by containerized or micro-VM based runtime environments. Platforms implementing this serverless model are categorized as Function as a Service (FaaS). Some examples of commercial serverless platforms are Amazon Web Services (AWS) Lambda, Google Cloud Functions or Microsoft Azure Functions, and there are also some open source serverless initiatives^23–25^, such as Apache OpenWhisk^26^, OpenLambda^27^, OpenFaas^28^ or Knative.

Cloud computing, and particularly the serverless computing paradigm, have been used for the deployment and implementation of scientific workloads in many recent works^29–33^. Scientific workflows are data-intensive workflows that require high computation and storage power^34^, and they include the fields astronomy, earthquake science, biology, and gravitational physics, among others^35^. One of the most important issues to handle the scientific workflows is to manage the resources and services of underlying computing platform. Scientific workflows management is a process in which cloud resources and services are procured to evaluate scientific application and then released accordingly. In this context, serverless has revealed itself as a valuable paradigm for the management of scientific workloads that demand optimal resource provision, since dynamic load peaks can effectively attended via automatic resource allocation and scaling. In this way, developers don’t have to worry about selecting, provisioning, and managing backend resources, but they only have to provide the application’s source code in the form of an atomic functions^36^. In addition, serverless platforms offer the possibility of function chaining^37–39^. This is a technique in which the output of one function is used as the input to another function. Function chaining can be used in conjunction with serverless computing to create powerful, event-driven workflows. By linking together multiple functions, it is possible to build complex applications that can respond to specific events in real-time.

In this work, we propose the use of the Amazon’s AWS Lambda serverless platform to deploy and implement our scientific application for Martian aurora detection. AWS Lambda provides an event-driven FaaS environment, which means that functions are invocated and ejecuted when a specific event or trigger occurs. This event could be a user uploading a file to an S3 bucket, a new item being added to a database, or a change in the state of an EC2 instance, among others. When the event occurs, AWS Lambda automatically runs the user’s code, processes the event, and scales the resources as needed.

### Proposed serverless architecture

Detecting auroras on Mars poses complexity and challenges, which require advanced imaging and analytical techniques. To address this, we developed a novel serverless architecture that uses Amazon Web Services and SageMaker, detecting Martian auroras with unmatched accuracy and speed.

**Figure 1:**
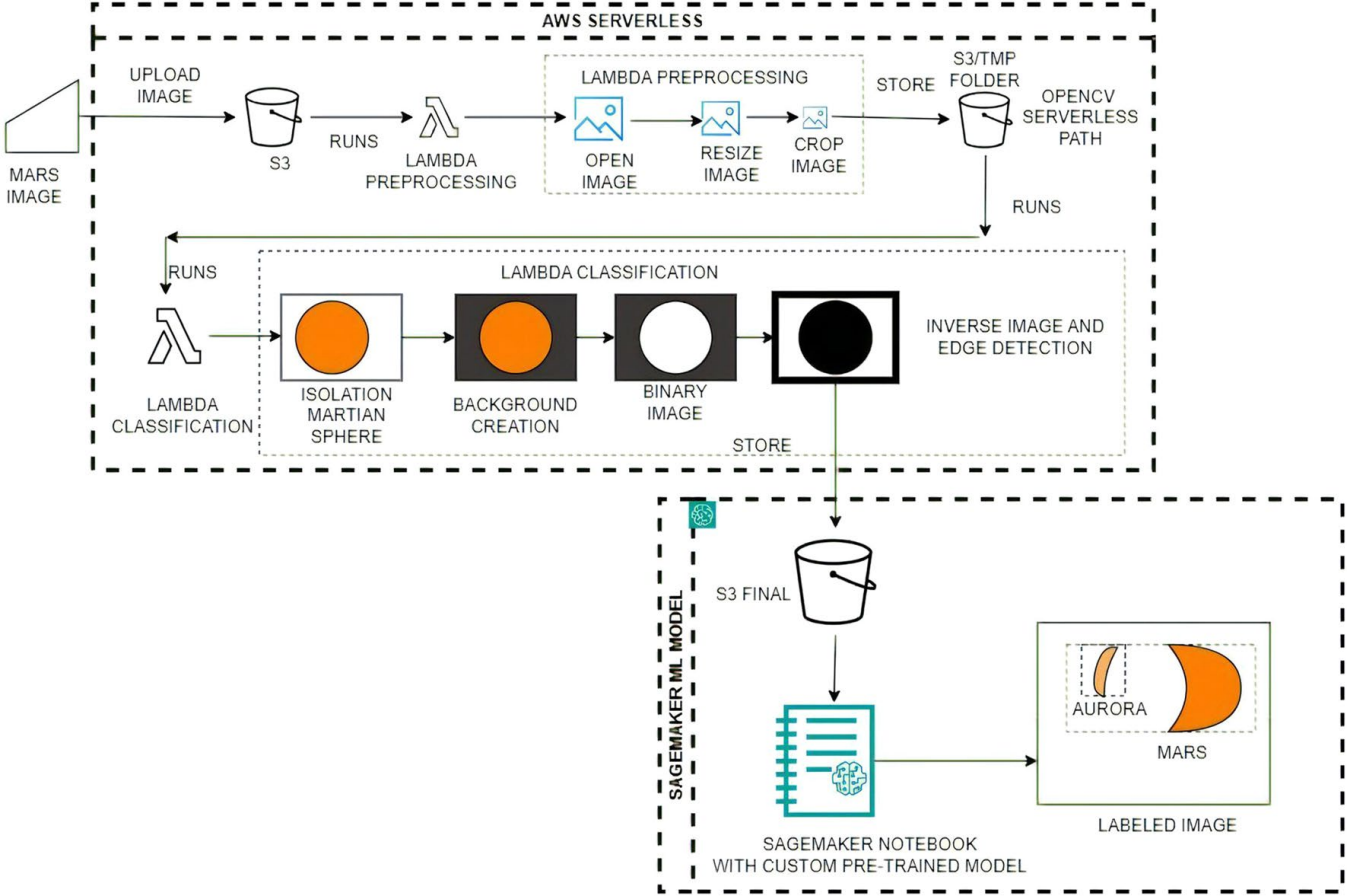
Distributed serverless architecture for Machine Learning inference, powered by our custom pre-trained model, and seamlessly executed in a SageMaker notebook.

Our proposed architecture (see Fig. 1) manages the vast data generated by Martian imaging missions while offering adaptability and scalability for evolving research needs. The core components of the architecture include a highly secure and reliable cold storage system in S3, dual Lambda functions that perform advanced image analysis using openCV, and a robust predictive machine learning model in SageMaker.

#### Lambda preprocessing function

In the following subsection, the specifics of the Lambda preprocessing function is presented. This function executes a series of steps aimed at transforming raw Mars data into a format suitable for further analysis. The steps are as follows:

1. **File opening and image generation:** The function begins by opening the raw data file with the fits.open() method, generating initial, non-standardized images of Mars atmospheric layers.
2. **Image resizing:** To standardize the images, they are resized to a uniform dimension. This ensures consistency across the data set and assists in subsequent analysis.
3. **Image cropping:** Lastly, the function crops the images to eliminate white spaces and center the Martian sphere. This step further refines the images, focusing attention on the most relevant areas.

This architecture (see Fig. 1) starts to generate images of Mars based on files of the emm_emu_l2b format, this process works on **Lambda Preprocessing**. Specifically, it extracts the necessary data from the file using the fits.open() function, part of the astropy.io.fits module, which plays a pivotal role in the processing of raw data related to Mars’ atmospheric layers. This function opens a FITS file, a standard data format widely used in astronomy, and prepares it for further processing. Once the FITS file is opened using fits.open(), the data can be accessed and manipulated using various methods. Then, the function processes it to obtain the logarithm of the energy value. This processed data is then used to create a heatmap using the Matplotlib library, with the resulting image being saved in PNG format.

**Figure 2:**
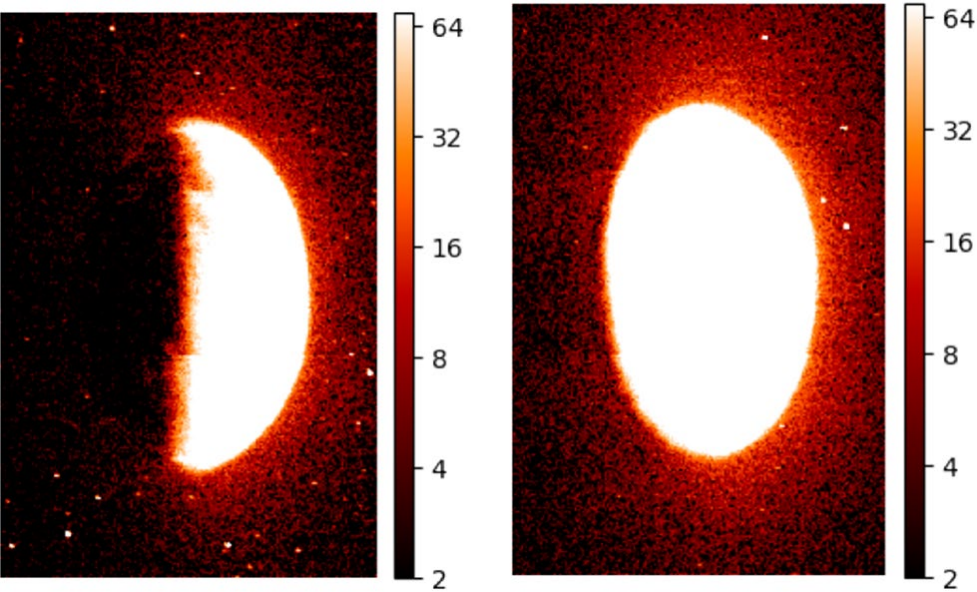
Heatmap of UV emission in the Martian atmosphere, as derived from emm_emu_l2b files. The logarithm of the UV intensity of the O 130.4 nm line was used to generate these images, providing valuable insights into the distribution of Oxygen in the Martian atmosphere and the detection of electron auroras on the nightside. The left image shows a transition to the nightside with the dark portion representing the shadowed nighside area of the planet with diminished UV emissions, while the right image presents a full view of the Martian sphere fully illuminated, indicative of the dayside atmosphere heavily influenced by solar UV flux. These contrasting visualizations exemplify the diurnal variations in Martian atmospheric UV activity and help in understanding the spatial distribution of oxygen and auroral phenomena.

It is worth noting that the generated images may have different sizes (see example at Fig. 2), as this depends on the original data and the specific parameters used in the processing and visualization steps. The resulting images can provide valuable information about the distribution and intensity of UV emissions from Oxygen in the Martian atmosphere, which can be useful for a variety of scientific purposes, such as detecting auroras or studying evolution of the planet’s atmosphere.

In order to accurately analyze and compare different images of Mars generated through the emm_emu_l2b processing and visualization code, it is important to ensure that they are all normalized to the same size and resolution.

**Figure 3:**
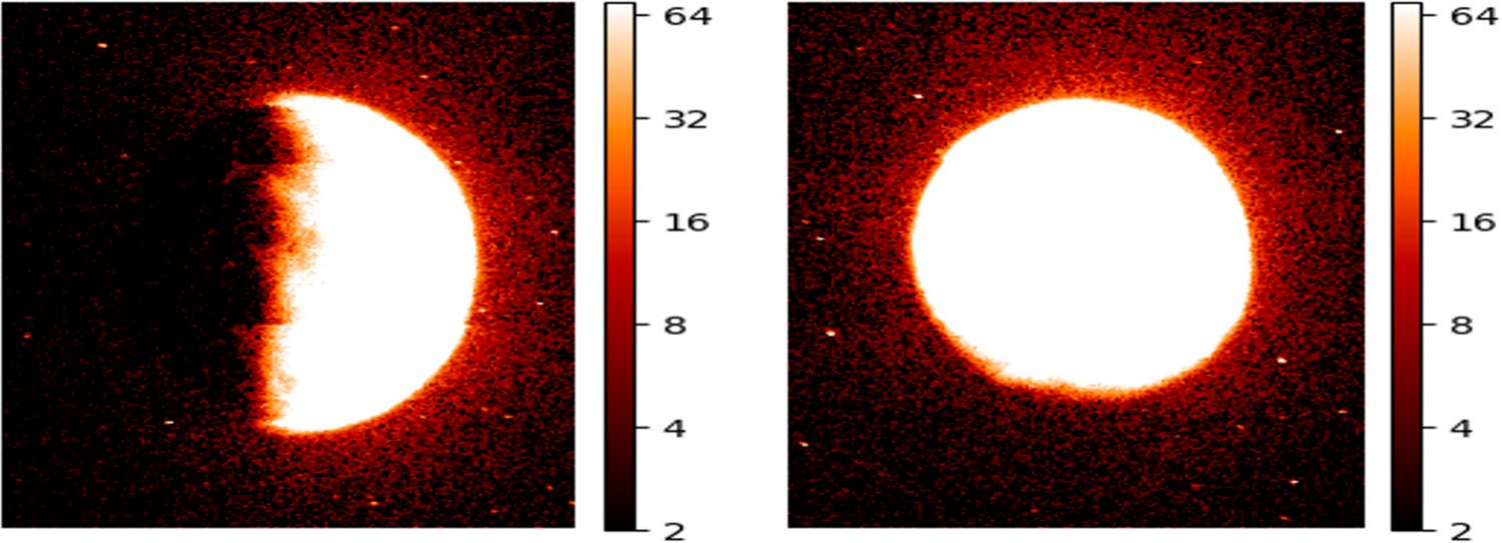
A normalized and resized image of the Martian atmosphere, generated using the emm_emu_l2b processing and visualization code and the AWS Lambda function shown earlier. The image has been resized to a standard size of 640$\times$480 pixels.

To achieve this normalization, the lambda function is triggered every time a new image is generated. The function opens the image file, resizes it to a standard size of 640$\times$480 pixels (see Fig. 3), and saves it in a new file location. This ensures that all images are of the same size and resolution, which simplifies subsequent analysis steps such as cropping and centering the Martian region of interest.

Once the resized image is generated and cropped, it is stored in another S3 bucket for further processing.

The objects in that S3 folder triggered our last Lambda function, The **Lambda Classification function** that is designed to identify images that contain “objects” within the Martian sphere, and eliminate those that do not.

**Figure 4:**
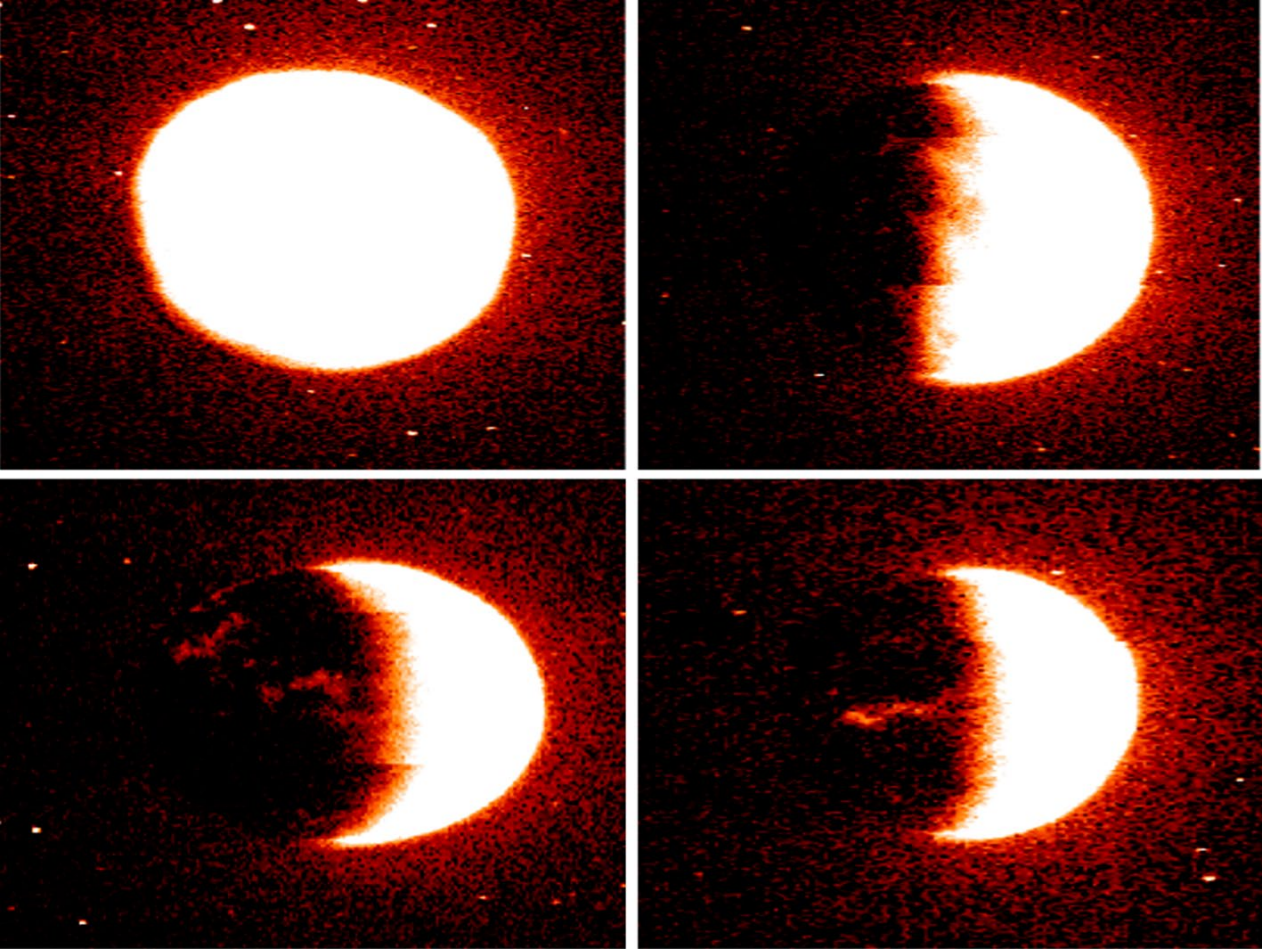
The cropped and resized image shows the selected region of interest (ROI) containing the Martian atmosphere, allowing for further analysis and detection of auroras using machine learning algorithms.

To achieve this, the function first crops the original image to remove any extraneous information outside the Martian sphere. It then resizes the resulting image to a standard size of 640$\times$480 pixels(see Fig.  4), to ensure consistency and accuracy in subsequent analyses. Finally, it saves the processed image to another S3 bucket, where it can be further analyzed and processed as needed.

#### Lambda classification function

The Lambda classification function, another critical component of our image analysis pipeline, carries out a series of transformations and operations that enable the detection and classification of objects within the Martian sphere. The function executes the following steps:

1. **Isolation of the martian sphere:** The function first isolates the Martian sphere within the image, focusing solely on the region of interest and reducing potential noise from the surroundings.
2. **Background creation:** A background for processing is then created, which involves the removal of the alpha layer. This simplification of the image aids in the subsequent processing steps.
3. **Binary image generation:** The function generates a binary image, an essential step for edge detection. By simplifying the image to two colors, the edges of objects become more apparent.
4. **Image inversion and edge enhancement:** Initially, the image is inverted. This manipulation enhances the edge detection of objects within the Martian sphere. Subsequently, the edge size is increased followed by a second inversion, which optimally detects the internal objects, thereby setting the stage for their classification.

This is an important function in the image processing pipeline, as it ensures that only the most relevant and informative images are passed on to the next stage of analysis. For this function, there are going to be a few steps to classify the martian images before Machine Learning inference.

In detail, this uses computer vision techniques to create a mask that highlights the area of interest in the image. The input image is read, and its dimensions are extracted. Then, a circular mask is defined with a radius of 170 pixels and its center located at (320, 230) pixels, which corresponds to the center of the Mars sphere. This mask is created by drawing a white circle on a black background.

**Figure 5:**
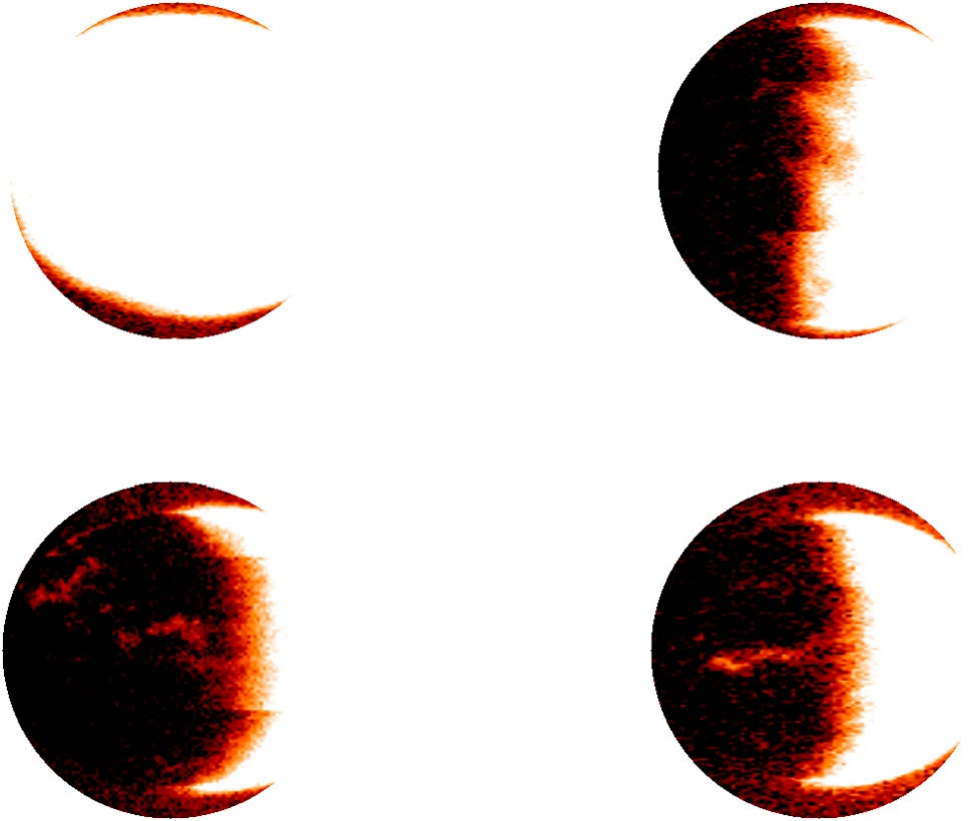
Preprocessed image of Mars surface with circular mask applied to remove non-object areas, generated using computer vision techniques in the AWS Lambda function. The circular mask is centered on the Mars sphere and only retains pixels within a certain radius.

Next, the mask created (see Fig.  5) is subtracted from the original image, resulting in a single channel image where only the pixels inside the circle are white and the rest are black. This mask is then added to the alpha channel of the original image, effectively making the pixels outside the circle transparent. Finally, the resulting image is saved in the tmp folder in the lambda function.

**Figure 6:**
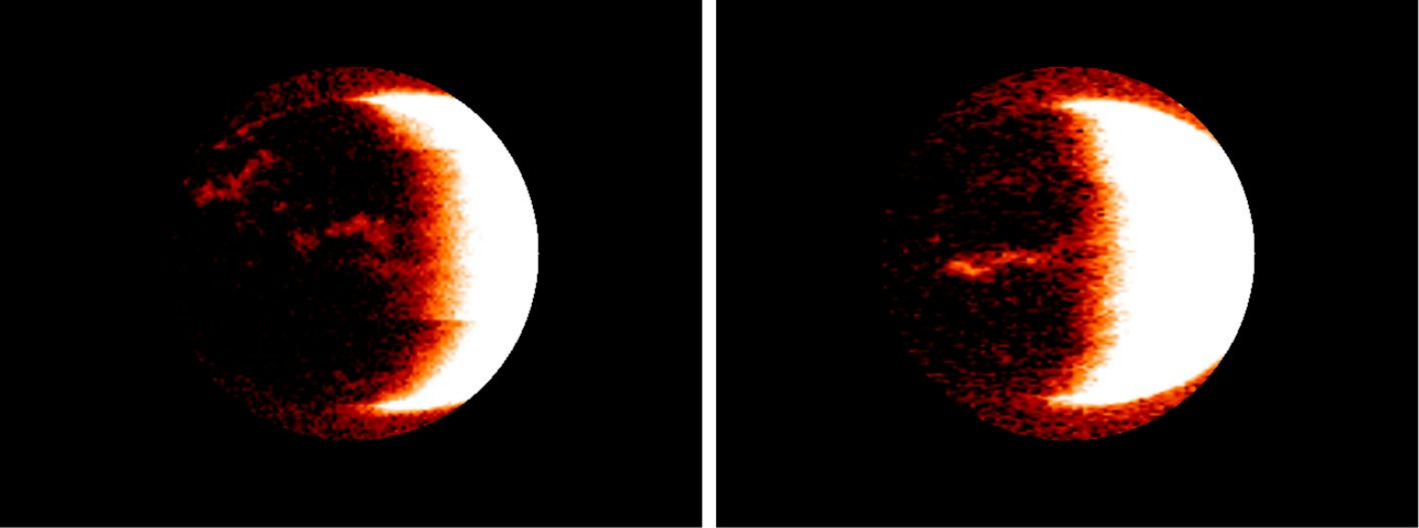
Preprocessed image of Mars surface with background applied.

By creating a mask that highlights the Mars sphere, the subsequence machine learning model can focus on analyzing only the relevant features of the image, which saves computational resources and improves the accuracy of the analysis (see Fig.  6), so these resulting figures will be almost ready for border detection using OpenCV.

**Figure 7:**
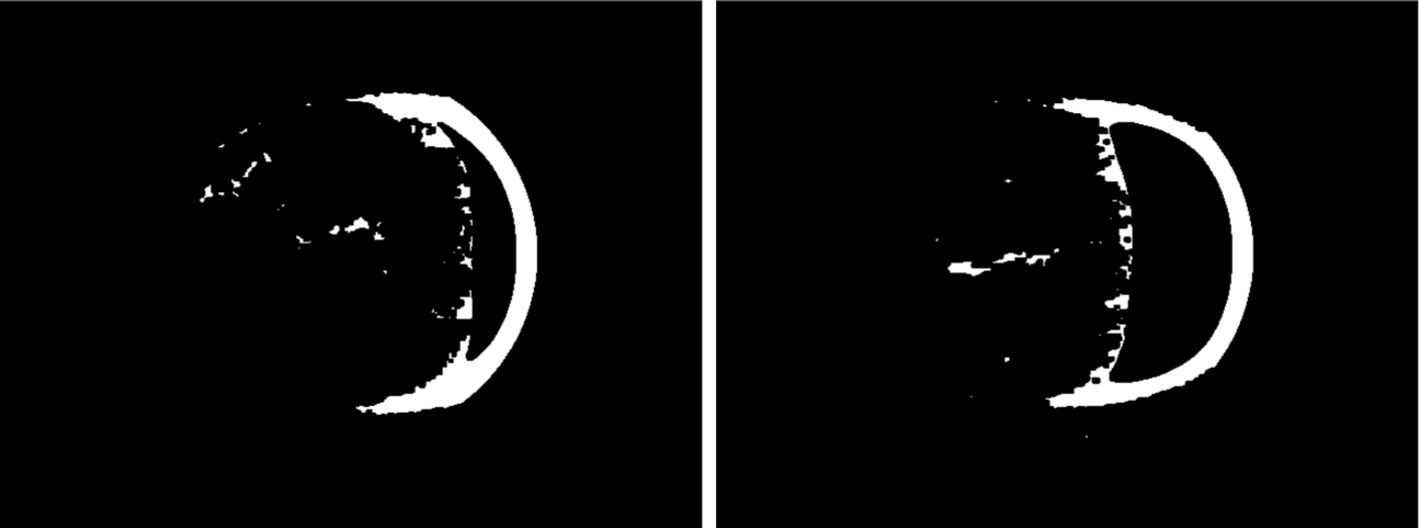
Binary image generated from preprocessed Mars surface image using adaptive thresholding and erosion operations. The resulting image highlights the areas with potential features or anomalies for further analysis.

In the next step, the classification function produces binary images for the border detection process (see Fig.  7). The function first converts the image from the BGR colorspace to a grayscale image. Then, it applies an adaptive threshold method to the grayscale image, which converts the image to a binary image, where each pixel is either black or white, based on a threshold value. This value is calculated by the adaptive threshold method (OpenCV).

After this, the function applies a morphological operation of erosion to the binary image, using a square kernel of size 4$\times$4 and the erode function (OpenCV). This erodes away the boundaries of the foreground object and reduces the size of the background pixels. The resulting image is stored on his S3 (S3 / TMP FOLDER) only if many objects are detected. Otherwise, the image is discarded, considering that it does not provide any valuable data inside the Martian circumference.

**Figure 8:**
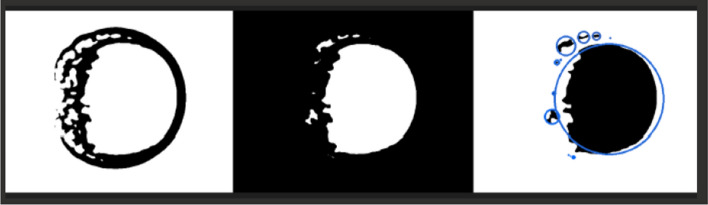
Image generated by the seed detection algorithm applied to the preprocessed image. The blue circles represent the detected objects, while the black regions correspond to the background. The top left panel shows the outlines of the objects, the top right panel shows the region where the flood-fill operation was applied to find the objects, and the bottom panel shows the detected objects with a circle around each one.

The final step of the classification function is very similar to the previous one, but using nearest detections. Its associated function converts the image from the BGR color space to grayscale, which reduces the information to one channel. Then it applies an adaptive threshold method to the grayscale image, converting it to binary using again the adaptive threshold method and then the erosion process (see Fig.  8).

**Figure 9:**
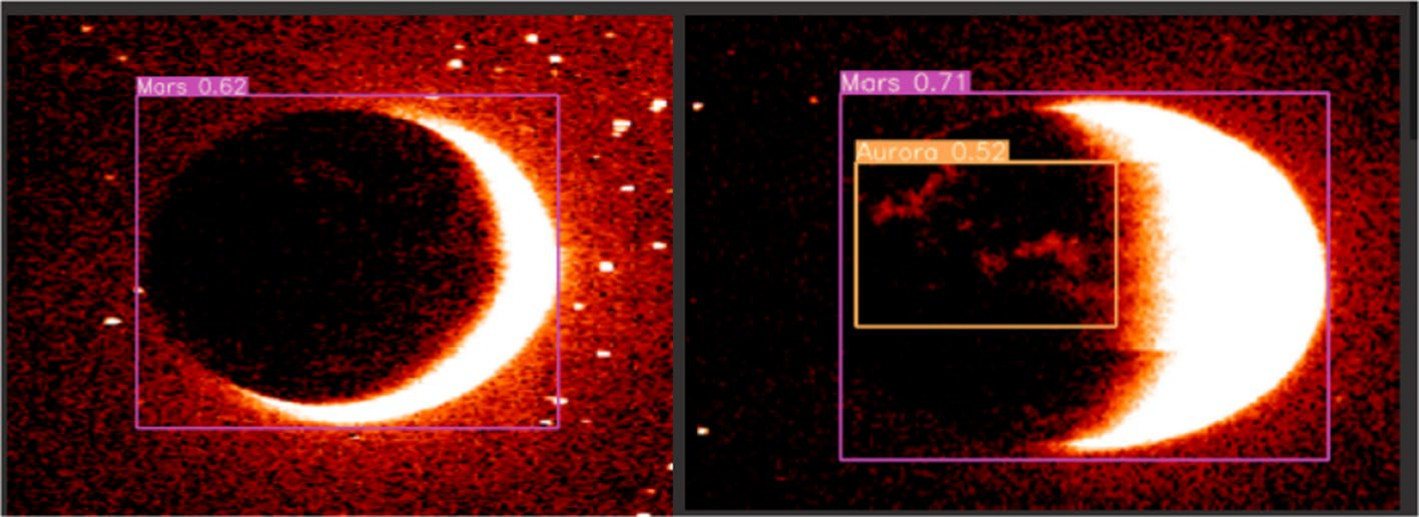
Final Machine Learning (SageMaker) detection, it shows Mars and auroras.

At this point, images not showing Martian auroras (images with less than two objects detected inside) can be safely discarded, reducing this way the overall execution time and cost. The rest are stored in a S3 bucket (S3 FINAL) and passed to the SageMaker process, where a pre-trained detection model is applied (see Fig.  9).

### Machine learning

Before delving into the intricacies of the SageMaker process, it is crucial to shed light on the foundational aspect of our endeavor - the creation of the training dataset.

#### Custom dataset for trainning

This dataset, formed using a sophisticated blend of interpolation algorithms and image processing techniques, is key to the successful operation of our machine learning models in the SageMaker environment. The process of dataset creation involves generating artificial Martian auroras, a feat achieved through a unique interplay of custom Python code and the Python Imaging Library (PIL).

**Figure 10:**
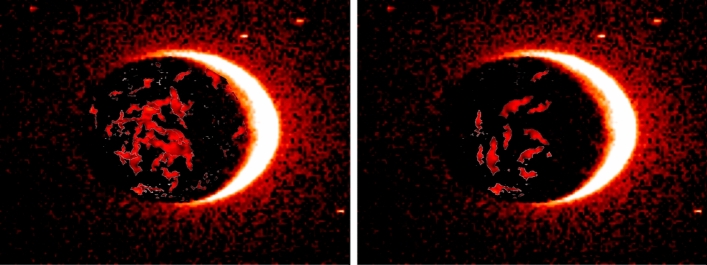
Examples of custom aurora for Machine Learning training generated by our custom model for making a complete for dataset.

As shown in Fig. 10, a custom aurora creation algorithm was created using 1000 synthetic images. Each image, a unique tapestry woven with varied aurora zones on Mars, reflects the distinct possibilities of aurora occurrences. These artificial constructs were seamlessly integrated with authentic images, creating a rich and diverse training dataset.

The task of training fell on the capable shoulders of YOLOv5, an object detection model renowned for its impressive performance in image detection tasks. YOLOv5 was chosen not just for its robust functionality but also for its proven compatibility within the SageMaker environment. This compatibility translates into seamless deployment and reliable performance, a key aspect considering the complexity of our dataset.

The choice of YOLOv5 is not without rationale. Its ability to distinguish the intricate features of our images, discerning the Martian planet from the aurora-representing spots, makes it an invaluable tool in our endeavor. Furthermore, its adaptability and efficiency align perfectly with our serverless architecture, ensuring a smooth and effective classification of Martian auroras despite the inherent scarcity of real-world data.

Once adequately trained, an ultimate test of the model using real world data was performed. Supplied by the HOPE mission, a collection of over 200 real images served as the proving grounds for our trained model. The results were not just encouraging, they were exceptionally promising.

The model detected the planet Mars with an astounding accuracy of 100%.

However, it’s essential to note that false positives were detected in images with low resolution or significant white areas.

Most importantly, such images would be filtered out in the serverless architecture before reaching the SageMaker phase, thereby eliminating potential sources of error. This crucial step ensures that the data fed into the SageMaker environment is of the highest quality, leading to more accurate and reliable results.

The initial date for the dataset under investigation is April 24th, 2021, while the final date is set at February 25th, 2022. By focusing on this specific time frame, we aim to provide a thorough and accurate evaluation of the EMM_EMU performance and trends during the aforementioned period.

#### Triggering SageMaker environment with the final dataset

With our training dataset and potential auroras residing in an S3 bucket named S3 FINAL, it triggers the SageMaker environment. This action sets in motion a process that runs the dataset through our trained model to perform aurora detection.

As the dataset navigates through the SageMaker environment, the model scrutinizes each image, searching for the telltale signs of an aurora. Upon successful detection, the image is promptly moved to a separate container.

This container serves as a repository for all detected aurora images, acting as a convenient collection point. These images can then be easily retrieved for further analysis, inspection, or dissemination.

This streamlined process ensures that aurora detection happens swiftly and accurately, with minimal human intervention. The use of SageMaker not only enhances efficiency but also facilitates scalability, accommodating larger datasets as our aurora detection endeavors expand.

## Experiments and results

### Validation of the serverless architecture

In our quest for a thorough assessment of our serverless architecture’s performance, we embarked on a series of experiments. We examined various image sets and different stack sizes, consistently observing rapid and efficient processing of large volumes of image data with minimal resource consumption.

Comparing the outcomes of image classification and machine learning inference in our serverless system to traditional server-based architectures allowed us to gauge accuracy. In numerous cases, our serverless setup displayed comparable or even superior results.

Stress tests were conducted, increasing image stack sizes and simulating high concurrent user traffic. Despite these challenges, our architecture maintained remarkable performance and accuracy levels.

We processed a 200-image stack through our system, employing AWS CloudWatch to measure OpenCV classification and machine learning inference durations. Averaging below 4 seconds for all 200 images, OpenCV classification consumed under 150 MB of RAM. Concurrently, machine learning inference was completed in under 40 seconds on average for the entire image stack.

These findings corroborate our serverless architecture’s capacity to swiftly analyze copious images with minimal resource utilization. The expeditious OpenCV classification signifies the system’s aptitude for real-time analysis. Though machine learning inference demanded more time, its performance remained laudable given the task’s intricacy.

Lastly, our system’s detection success rate surpassed 95%, demonstrating its efficacy in identifying and classifying image features. This exceptional accuracy enables seamless integration into our machine learning dataset, further enhancing system performance.

### Platform comparison and serverless architecture experimental results

#### Introduction to experimental setup

As mentioned before, the main goal of this experimental section is to compare the proposed serverless architecture with a traditional server-based solution. For this purpose, we also carried out different experiments in an on-premises computer using exclusively a single-threaded approach. This choice is motivated by the inherent complexity and challenges associated with implementing and managing multithreading in Python. Consequently, the potential advantages of multithreading will not be explored in this context. Furthermore, the number of times that Lambda functions can be parallelized, designated as ’*n*’, will be set to 1000. This number is chosen to align with the contractual limit set for parallelization during the testing phase of our project. This constraint facilitates a more controlled and consistent evaluation environment, ensuring a fair comparison between the on-premises computer and our proposed AWS-based system.

#### Experiment design and dataset

The experiment was conducted utilizing the 200 real-world images provided by the HOPE mission. Both on-premises computer and AWS Lambda environments were used for processing these images to facilitate an accurate comparison of performance. However, the results presented will primarily focus on the figures obtained from 1000 Lambda function invocations. This focus is dictated by the set limit for Lambda parallelization; the processing times for 200 images and 1000 images remain comparable due to the inherent scalability of Lambda functions. This approach allows us to extrapolate and generalize our findings, providing a comprehensive view of the system’s performance when operating at its maximum parallelization capacity.

#### Cost analysis and performance comparison

In this section, we provide a comprehensive overview of the cost-effectiveness and performance of our system based on Amazon Web Services (AWS) compared to an on-premises computer. The cost for invoking the SageMaker function is $0.1345 per on-demand ml.m5.large instance-hour, while a Lambda function costs $0.00001667 per GB-second of computation time.

The total costs and execution times for processing 1,000 images using both AWS Lambda and an on-premises computer are compared in Table 1.

**Table 1:** Comparison of total processing times and costs for 1,000 images using an on-premises computer and AWS Lambda.

	On-premises computer	AWS Lambda
Total time for 1,000		
Images (seconds)	34,000	42
Total cost for 1,000		
Executions / machine cost (USD)	3,000 (Total PC prize)	0.02937 (Per 1000 exec.)

## Discussion

This study introduces a serverless computing framework for analyzing Martian auroras, utilizing AWS Lambda and Amazon SageMaker. Our approach, centered around a synthetic dataset, addresses the scarcity of Martian aurora observations, paving the way for high-precision machine learning models. These models, trained on a blend of real and artificial images, demonstrate advanced capability to accurately identify the unique features of Martian auroras.

This research offers significant knowledge into the atmospheric conditions of Mars in the wider context of space exploration. It complements existing studies by offering a new perspective on aurora analysis through cloud computing. However, challenges such as the limitations of synthetic datasets in capturing the full range of real-world variances need acknowledgment. These limitations could impact the generalizability of our findings and highlight the need for continuous enhancement of the dataset. We chose AWS Lambda and Amazon SageMaker due to their scalability, efficiency, and capacity to manage large-scale image data. These tools offer a flexible and cost-effective solution for complex computational tasks, setting a benchmark for future studies in astronomical data analysis.

Exploring potential approaches for future research, it is possible to improve the serverless architecture for real-time data analysis and broaden its use to other celestial bodies. In addition, it would be beneficial to incorporate a wider range of data sets and refine the machine learning algorithms. The implications of this study for the field of space science are significant. By demonstrating the effectiveness of a cloud-based and serverless approach, this research opens new opportunities for more agile and scalable methods in astronomical data analysis. This could be a significant addition to the ever-changing field of remote sensing and the exploration of celestial objects, offering a model for future research works.

## Conclusions

The research has effectively shown that serverless computing is able of examining Martian auroras, forming a framework that maximizes both productivity and analytical complexity. We are looking to progress our research by combining an Amazon Elastic File System (EFS) container with AWS Lambda and Amazon API Gateway. This integration will facilitate a comprehensive comparison with our existing serverless architecture, focusing on aspects such as performance, cost-efficiency, scalability, and reliability.

By incorporating Amazon EFS, we anticipate a significant enhancement in handling extensive image datasets, crucial for in-depth aurora analysis. This step is expected to further streamline data processing and management, contributing to the robustness of our research methods. The outcome of this exploration will not only refine our current approaches, but also provide valuable insights into optimal computational architectures for space science research. This initiative aligns with our commitment to advancing the field of Martian studies through innovative technological solutions, thereby contributing meaningfully to the broader scientific community’s understanding of Martian atmospheric phenomena.

## Data Availability

The software underpinning the research results of this study is available via Figshare with the following Digital Object Identifier (DOI): https://doi.org/10.6084/m9.figshare.23566296. This includes detailed descriptions of the system architecture, integrated components, and functionality. However, please note that certain elements of the software, such as the trained dataset and serverless version, are currently pending patent approval and, thus, have not been made publicly accessible at this time. As soon as these components receive patent approval, they will be made accessible in accordance with patent laws and regulations. The images used for the study were derived from various Mars missions, including the Emirates Mars Mission, and have been processed using the above-mentioned software. These processed data have been cited within the paper and can be accessed using the provided DOI.
